# The Therapeutic Potential of Zinc-Alpha2-Glycoprotein (AZGP1) in Fibrotic Kidney Disease

**DOI:** 10.3390/ijms23020646

**Published:** 2022-01-07

**Authors:** Inga Sörensen-Zender, Song Rong, Hermann Haller, Roland Schmitt

**Affiliations:** Department of Nephrology and Hypertension, Hannover Medical School, Carl-Neuberg-Strasse 1, 30625 Hannover, Germany; rong.song@mh-hannover.de (S.R.); haller.hermann@mh-hannover.de (H.H.); Schmitt.Roland@mh-hannover.de (R.S.)

**Keywords:** AZGP1, chronic kidney disease, kidney fibrosis, renal lipid metabolism

## Abstract

Chronic kidney disease (CKD) is characterized by a long-term loss of kidney function and, in most cases, by progressive fibrosis. Zinc-alpha2-glycoprotein (AZGP1) is a secreted protein, which is expressed in many different tissues and has been associated with a variety of functions. In a previous study, we have shown in cell culture and in AZGP1 deficient mice that AZGP1 has protective anti-fibrotic effects. In the present study, we tested the therapeutic potential of an experimental increase in AZGP1 using two different strategies. (1) C57Bl/6J mice were treated systemically with recombinant AZGP1, and (2) a transgenic mouse strain was generated to overexpress AZGP1 conditionally in proximal tubular cells. Mice underwent unilateral uretic obstruction as a pro-fibrotic kidney stress model, and kidneys were examined after 14 days. Recombinant AZGP1 treatment was accompanied by better preservation of tubular integrity, reduced collagen deposition, and lower expression of injury and fibrosis markers. Weaker but similar tendencies were observed in transgenic AZGP1 overexpressing mice. Higher AZGP1 levels led to a significant reduction in stress-induced accumulation of tubular lipid droplets, which was paralleled by improved expression of key players in lipid metabolism and fatty acid oxidation. Together these data show beneficial effects of elevated AZGP1 levels in fibrotic kidney disease and highlight a novel link to tubular cell lipid metabolism, which might open up new opportunities for CKD treatment.

## 1. Introduction

Chronic Kidney Disease (CKD) is a condition characterized by a gradual loss of kidney function over time. It is typically accompanied by progressive fibrosis of the tubulointerstitial compartment [1]. In previous studies, we identified zinc-alpha2-glycoprotein 1 (AZGP1) as an important factor in attenuating the development of kidney fibrosis [2,3]. AZGP1 is a secreted glycoprotein that is synthesized by adipocytes and epithelial cells of many organs. It can be found in different body fluids like serum, saliva, sweat, milk, and urine [4,5]. AZGP1 protein (41–43 kDa) is freely filtered in the glomerulus and subsequently cleared by kidney tubular cells [6]. Therefore, circulating AZGP1 significantly accumulates in patients with disrupted renal function and acute or chronic renal failure [3,7]. Similar to other organs, kidney fibrosis is characterized by dedifferentiation of epithelial cells and activation of interstitial fibroblasts. AZGP1 has been shown to promote epithelial cell differentiation and counteract TGF-β induced fibroblast activation [2,8,9,10]. AZGP1 also plays an important role in lipid metabolism and cancer cachexia, where it has been described as a lipid-metabolizing factor [11]. Mice that were treated with recombinant AZGP1 showed increased fat mobilization, whereas genetic deletion of AZGP1 was associated with increased body fat [12,13,14]. In the liver, different studies showed a significant impact of AZGP1 on fatty acid metabolism, such as accelerated lipolysis and reduced inflammation in non-alcoholic fatty liver disease (NAFLD) [15,16,17,18].

Here, we tested new opportunities for the therapeutic potential of AZGP1 using murine unilateral ureteral obstruction (UUO) as a fibrotic kidney disease model and evaluated potential implications of AZGP1 in renal lipid metabolism. 

## 2. Results

### 2.1. Administration of Recombinant AZGP1 Attenuates Renal Fibrosis Development

**Figure 1:**
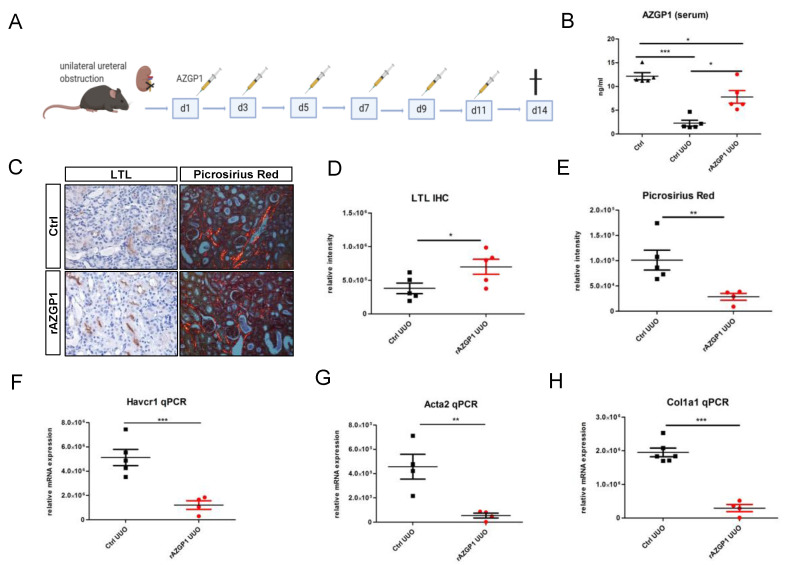
(**A**): Experimental design for the treatment with recombinant AZGP1 (rAZGP1) after UUO. (**B**): AZGP1 levels in serum of untreated mice without surgery and untreated and treated mice 14 days after UUO. (**C**): Lotus tetragonolobus lectin (LTL) staining and Picrosirius Red staining after 14 days UUO. (**D**). Quantification of LTL staining. (**E**): Quantification of Picrosirius Red staining. (**F**–**H**): Damage and fibrosis marker mRNA expression after 14 days UUO. Significance was tested by student’s *t*-test. * *p* < 0.05; ** *p* < 0.01; *** *p* < 0.001.

On the basis of our previous data [2,19], we hypothesized that exogenous administration of AZGP1 may inhibit fibrotic processes in the kidney. To test this, we induced experimental renal fibrosis by UUO in male C57Bl/6J mice treated with recombinant AZPG1 (rAZGP1) or with vehicle. After surgery, mice were injected intravenously with rAZGP1 (150 µg per injection) or vehicle every other day until day 11 (Figure 1A). On day 14, kidneys were harvested, and blood was taken to determine AZGP1 serum levels. Three days after the last injection, serum AZGP1 levels were still significantly higher in rAZGP1 treated mice (Figure 1B). This was accompanied by better preservation of proximal tubular integrity as shown by Lotus tetragonolobus lectin (LTL) binding (Figure 1C,D) and by reduced collagen deposition as shown by Picrosirius Red staining (Figure 1C,E). In parallel, we observed significantly lower expression of Havcr1 (coding for kidney injury marker KIM-1), Acta2 (coding for the fibroblast activation marker α-smooth muscle actin; αSMA), and Col1a1 (coding for collagen type I) in kidneys of AZGP1 treated mice (Figure 1F–H). Together, these data indicated anti-fibrotic effects of rAZGP1 administration in kidneys challenged by UUO stress. 

### 2.2. Transgenic AZGP1 Expression in Proximal Tubule Provides Protection in UUO

**Figure 2:**
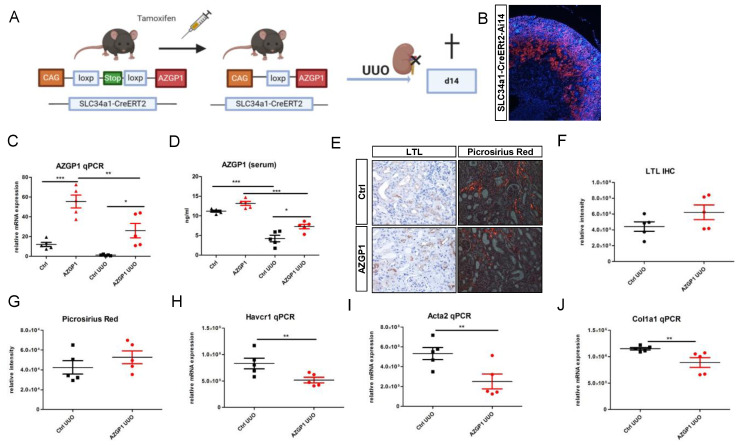
(**A**): Schematic of transgenic mouse model for conditional overexpression of AZGP1 in the proximal tubule. (**B**): tdTomato positive tubules showing Ai14 reporter expression of successful Cre recombination after tamoxifen administration. (**C**): AZGP1 mRNA expression levels in control and UUO kidneys at 2 weeks after tamoxifen treatment. (**D**): AZGP1 serum levels of AZGP1 overexpressing mice as measured by ELISA. (**E**): LTL staining and Picrosirius Red staining after 14 days of UUO. (**F**): Quantification of LTL staining (**G**): Quantification of Picrosirius Red. (**H**–**J**): Damage and fibrosis marker mRNA expression after 14 days UUO. Significance was tested by student’s *t*-test. * *p* < 0.05; ** *p* < 0.01; *** *p* < 0.001.

To consolidate the observed effects by an independent method, we generated a mouse model with transgenic AZGP1 expression driven by a proximal tubule-specific promoter. Transgenic mice carrying murine Azgp1 cDNA with an upstream loxP-Stop-loxP sequence were crossed with Slc34a1-CreERT2 mice to achieve tamoxifen-inducible AZGP1 overexpression in proximal tubular cells (Figure 2A). Successful Cre recombination in proximal tubules was confirmed by additional tdTomato reporter gene co-expression (Figure 2B). Tamoxifen-induced overexpression of AZGP1 resulted in a mean 4,6-fold increase of Azgp1 transcript levels in control kidney homogenates. (Figure 2C). This increase in healthy control mice was smaller than anticipated and was only associated with a small but non-significant increase in serum AZGP1 (Figure 2D). Tamoxifen-induced transgenic mice and control mice underwent UUO surgery, and kidneys were examined after two weeks. AZGP1 transcript, as well as serum levels, showed a significant decrease after UUO (Figure 2C,D). While we found no significant differences in tubular LTL binding or Picrosirius Red staining in the AZGP1 overexpressing mice (Figure 2E–G), we observed significantly reduced up-regulation of Havcr1, Acta2, and Col1a1 transcripts in the tamoxifen-treated group (Figure 2H–J). These data are consistent with a protective effect of transgenic tubular AZGP1 expression, although the observed differences were smaller as compared to the effects obtained with systemic rAZGP1 treatment. 

### 2.3. AZGP1 Reduces Intrarenal Lipid Accumulation and Acts as an Inducer of Key Players in Lipid Metabolism

**Figure 3:**
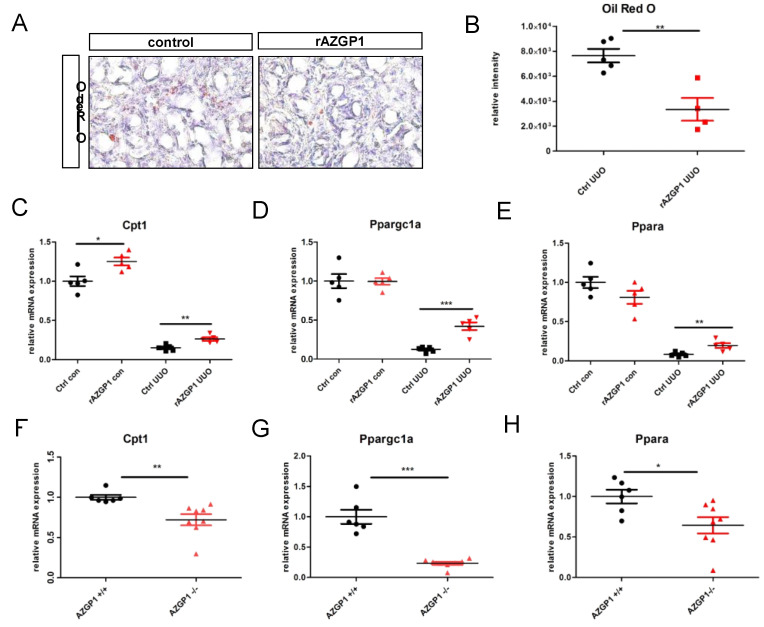
(**A**): Representative pictures of Oli Red O staining on rAZGP1 treated and control kidney cryosections after 14 days of UUO. (**B**): Quantification of Oil Red staining. (**C**–**E**). mRNA expression of carnitine palmitoyl-transferase 1A (Cpt1), PPARgamma coactivator-1a (Ppargc1a), and peroxisome-proliferator-activated receptor α (Ppara) in kidneys of AZGP1 treated mice. (**F**–**H**): mRNA expression of Cpt1, Ppargc1a, and Ppara in AZGP1 deficient mice. Significance was tested by student’s *t*-test. * *p* < 0.05; ** *p* < 0.01; *** *p* < 0.001.

We and others have previously linked anti-fibrotic activities of AZGP1 to TGF-beta antagonizing mechanisms and reduced fibroblast activation [2,20,21]. Additional mechanisms, in particular a potential involvement of AZGP1 in altered lipid metabolism of the kidney, have not been explored. Given its properties as a lipid degradation factor, we evaluated changes in lipid accumulation in UUO kidneys. Quantification of Oil Red O staining revealed a significant reduction of tubular lipid droplet deposition in rAZGP1 treated kidneys (Figure 3A,B). Concomitantly, the expression of key players of tubular fatty acid oxidation (FAO) was significantly different between the groups. Quantitative PCR revealed elevated transcripts for carnitine palmitoyl-transferase 1A (Cpt1), peroxisome-proliferator-activated receptor α (Ppara), and PPARgamma coactivator-1a (Ppargc1a) in contralateral (con) as well as ligated (UUO) rAZGP1 treated kidneys (Figure 3C–E). These data suggested that AZGP1 plays a role in stabilizing renal tubular lipid balance and FAO during stress conditions. Accordingly, we observed an inverse pattern in kidneys from AZGP1 knockout mice, which showed a significantly decreased expression of Cpt1, Ppara, and Ppargc1a (Figure 3F–H).

### 2.4. Altered Lipid Metabolism in AZGP1 Deficient Tubular Cells 

**Figure 4:**
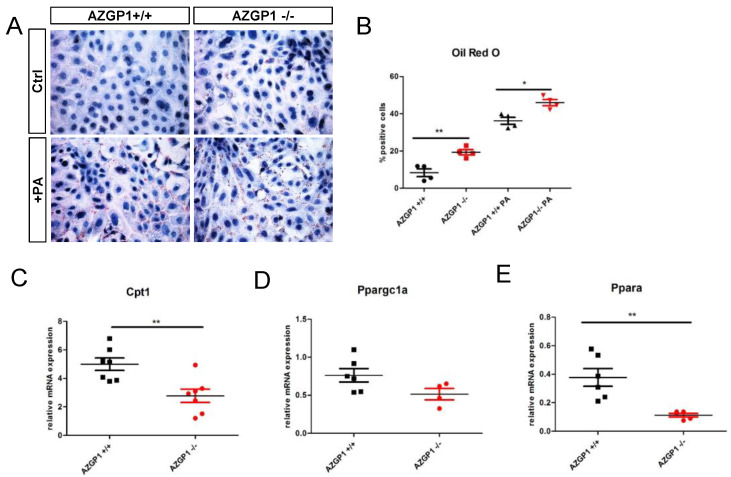
(**A**): Oil Red O staining in primary tubular epithelial cells (PTEC) isolated from wildtype or AZGP1 deficient mice untreated or treated with palmitic acid. (**B**): Quantification of Oil Red O staining in PTEC. (**C**–**E**): mRNA expression of Cpt1, Ppargc1a, and Ppara in wildtype and AZGP1 deficient PTEC. Significance was tested by student’s *t*-test. * *p* < 0.05; ** *p* < 0.01.

To further differentiate primary effects of AZGP1 from secondary changes due to UUO, we analyzed isolated primary tubular epithelial cells (PTEC) from kidneys of AZGP1 knockout and wildtype mice. In AZGP1 deficient PTEC, we observed increased lipid droplets by Oil Red O staining (Figure 4A,B). The increased lipid accumulation was accentuated when PTEC were challenged with 0.4 mM palmitic acid (PA) for 24 h (Figure 4A,B). On the transcriptional level, knockout PTEC showed significantly lower levels of Cpt1 and Ppargc1a, as well as a trend for lower Ppara expression (Figure 4C–E). Together, these results provide evidence for the involvement of AZGP1 in tubular epithelial lipid metabolism. 

## 3. Discussion

In this study, we tested the therapeutic potential of AZGP1 in the treatment of pro-fibrotic CKD. C57Bl/6J mice were treated with recombinant AZGP1, and a new transgenic mouse strain was generated with conditional overexpression of AZGP1 in proximal tubular cells. Both strategies were associated with attenuation of the pro-fibrotic mechanism, supporting the hypothesis that AZGP1 might be useful in antagonizing CKD progression. 

Mechanistically, we show for the first time that AZGP1 has regulatory functions in kidney lipid metabolism. The importance of balanced fatty acid degradation in the development of fibrosis has been shown in different organs like the lung, liver, and kidney [16,22,23,24]. In tubular epithelial cells, lipid accumulation and defective FAO have been recognized as key mechanisms in promoting kidney fibrosis [24]. We found that UUO induced lipid accumulation was attenuated by rAZGP1 and that the expression of genes centrally involved in lipid transport and FAO was better maintained despite UUO stress. Transcription of CPT1, which is required for fatty acid transport into the mitochondria, was significantly higher in rAZGP1 treated mice, whereas it was reduced in AZGP1 deficient kidneys. Yuan et al. recently demonstrated that genetic ablation of CPT1 aggravated tubular injury and promoted interstitial fibrosis [25]. Accordingly, Miguel et al. found that the development of kidney fibrosis could be efficiently rescued by overexpressing CPT1 in tubular cells [23]. These findings clearly highlight important anti-fibrotic properties of CPT1 and suggest that AZGP1 dependent stabilization of CPT1 expression might contribute to the observed benefits in our experiments.

Besides CPT1, we found better preservation of Ppara and Ppargc1a expression in rAZGP1 treated kidneys. Both are key transcription factors that regulate the expression of different proteins involved in fatty acid uptake and FAO (including CPT1) [26,27]. Expression of Ppara and Ppargc1a is known to be disrupted in human CKD and in animal models of kidney fibrosis [28,29,30,31]. In a study by Kang et al., it was demonstrated that Pparcg1a overexpression reduced pro-fibrotic changes while normalizing the expression of CPT1 [24]. Interestingly, the authors of the same study observed that TGF-β is acting as an upstream repressor of Ppargc1a via direct Smad3 binding to the gene regulatory region [24]. Based on our previous findings that AZGP1 can act as a TGF-β pathway inhibitor [2], it is possible that TGF-β dependent effects are involved in the observed lipid regulatory processes. This is an aspect we want to analyze in future studies, as it could lead to novel anti-fibrotic pathways at the intersection between AZGP1, TGF-β, and PPARs [32]. 

Our study has several shortcomings. First, while the UUO model is widely used to study CKD and renal fibrosis, it does not allow studying the impact of anti-fibrotic agents, e.g., AZGP1, on renal function. To this end, a reversible UUO model would have been necessary [33]. Second, the anti-fibrotic effect size of transgenic AZGP1 overexpression was only moderate as compared to systemic rAZGP1 treatment. There are several potential explanations for this. (1) transgenic AZGP1 expression was weaker than anticipated. In kidney homogenates, we only found a 4.3-fold increase of transcript, and we only found a small but non-significant increase in systemic protein levels. Quantification of intrarenal protein abundance was unfortunately not possible due to lack of reliable antibodies for murine tissues; (2) we used a proximal tubule-specific promoter to drive transgenic AZGP1 expression. Local restriction of AZGP1 expression might have weakened the mechanism of action because UUO damage affects the entire kidney; (3) systemically administered circulating rAZGP1 might have extrarenal effects (e.g., on immune cells), which alter the course of UUO damage. Future plans include crossing our transgenic mice to other Cre-recombinase strains to obtain stronger and more robust conditional overexpression of local and systemic AZGP1. While this will allow us to obtain additional insight, the current results already provide clear signals for a relevant activity of AZGP1.

In summary, our data indicate that therapeutic elevation of AZGP1 has beneficial effects by targeting the development of kidney fibrosis. The precise role of the involvement of AZGP1 in kidney lipid metabolism warrants further investigation and might open up new opportunities for the treatment of CKD. 

## 4. Material and Methods

### 4.1. Transgenic Mice

To generate transgenic mice, a PiggyBAC based strategy (Cyagen Biosciences, Santa Clara, CA, USA) was used for pro-nucleus microinjection of murine Azgp1 cDNA with an upstream loxP-Stop-loxP sequence and a CAG promoter. A mouse strain with the genetic background C57Bl/6N was used to generate this transgenic line. Founder pups were identified with polymerase chain reaction (PCR) by using transgene-specific primers, and offsprings were mated with Slc34a1tm1^(EGFP/cre/ERT2)Bhum^ mice (kindly provided by Dr. B. Humphreys, Washington University School of Medicine, St. Louis, MO, USA). These mice contain a CreERT2 cassette in the SLC34a1 locus, which enables selective expression of the Cre recombinase in proximal tubules after tamoxifen injection [34]. Overexpression of AZGP1 in proximal tubules was induced 14 days before UUO surgery by tamoxifen (Carbolution Chemicals GmbH, St. Ingbert, Germany) administration, i.p., every other day (20 mg/mL) for 4 times in AZGP1-SLC34a1-CreERT2 mice. To test for successful Cre-recombination, AZGP1 mice were crossed with Ai14 reporter mice (B6.Cg-Gt*(ROSA)*26Sor^tm14(CAG-tdTomato)Hze^/J, Jackson Laboratory, Bar Harbor, ME, USA). 

### 4.2. Unilateral Ureteral Obstruction (UUO)

Male wildtype mice (C57BL/6J purchased from Janvier Labs (Le GenestSaintIsle, France)) and transgenic AZGP1-SLC34a1::CreERT2 mice treated with tamoxifen or vehicle were subjected to UUO at age 10–12 weeks. Mice were anesthetized with isoflurane and underwent left proximal ureteral ligation via a midline abdominal incision. Mice were killed, and the kidneys were analyzed 14 days after UUO surgery. Wildtype mice were treated with systemic injections of recombinant AZGP1 (150 µg i.v.) or vehicle (PBS) every other day for the duration of the experiment. All experimental procedures were in agreement with institutional and legislative regulations and approved by the local authorities. 

### 4.3. Cell Culture 

Primary renal tubular epithelial cells (PTEC) were isolated as previously described [35] and grown in REGM2 (PromoCell, Heidelberg, Germany). Cells were treated on day 6 of culture with recombinant AZGP1 (25 µg/mL) and/or Palmitic acid (0.4 mM, Sigma-Aldrich, St. Louis, MO, USA) for 24 h. 

### 4.4. Histological and Immunohistochemical Stainings

Obstructed and contralateral kidneys were dissected, fixed in 4% PFA, and embedded in paraffin for cryosectioning. Four-micrometer paraffin sections were used for Picrosirius Red staining and immunohistochemistry. Deparaffinized kidney sections were boiled in Tris/EDTA buffer for antigen-retrieval, blocked with 5% milk, and incubated overnight with biotinylated Lotus tetragonolobus lectin (LTL, Vectorlaboratories, Servion, Schwitzerland). This was followed by visualization with the ABC Vectastain kit (Vector Laboratories, Burlingame, CA, USA).

Picosirius Red staining: Deparaffinized kidney sections were stained in Picosirius Red staining solution (Sigma-Aldrich, St. Louis, MO, USA) for 1 h, washed in acidified water, and mounted. The staining was visualized under polarised light, and birefringence was analyzed. Picosirius Red and LTL staining were quantified using Image J (NIH).

### 4.5. Oil Red O Staining

Oil Red O staining was performed using a staining kit (ScyTek, Logan, UT, USA) according to the manufactures instructions. Prior to the staining, cells or cryosections were fixed in 4% PFA for 15 min. Cryosections were analyzed using Image J (NIH). 

### 4.6. AZGP1 Measurement in Serum

AZGP1 (ng/mL) was measured in serum samples or kidney lysates with a commercial enzyme-linked immunosorbent assay (Novus Biologicals, Bio-Techne GmbH, Wiesbaden, Germany), according to the manufacturer’s instructions. 

### 4.7. Synthesis and Purification of AZGP1

HEK 293F cells were seeded in FreeStyle Medium (Gibco Invitrogen, Karlsruhe, Germany) and transfected with mammalian expression vector pcDNA3.1 containing mouse AZGP1. The medium was harvested every third day in 14 days of culture. The medium was centrifuged at 800× *g* for 15 min to remove cells and concentrated using an Ultra-15 centrifugal filter (Amicon, Billerica, MA, USA) with a 10-kDA cut-off. The concentrated medium was added to 1 g/10 mL diethylaminoethyl cellulose (DEAE, Sigma-Aldrich, St. Louis, MO, USA) in 10 mM Tris (pH 8.8) and stirred for 2 h at 4 °C. The negatively charged AZGP1 binds to DEAE-cellulose, which was sedimented by centrifugation and eluated by adding 10 mM Tris/0,3M NaCl and incubation for 30 min at 4 °C with stirring. The supernatant was concentrated using Amicon centrifugal filters (Amicon, Billerica, MA, USA) with a cut-off of 30-kDa. Protein concentration was measured using Bradford Assay.

### 4.8. Quantitative Real-Time PCR

RNA was isolated from PTEC and frozen kidney tissue using Nucleospin RNA Plus (Machery-Nagel, Düren, Germany) according to the manufactures instructions. Reverse transcription was performed with M-MLV-Reverse Transcriptase (Promega, Madison, WI, USA). Amplified cDNA was used as a template for qPCR. The levels of mRNA expression were determined by quantitative real-time PCR using a Roche Lightcycler 480 System with SYBR green master mix and specific primers. Melting curves were examined to verify that a single product was amplified. For quantitative analysis, all samples were normalized to Actin gene expression using the ▲▲CT value method. For primer sequences, see Table 1. 

### 4.9. Statistical Analysis

Results are expressed as means ± SEM. Statistical significance was assessed by unpaired *t*-test or by Log-rank tests performed on Kaplan–Meier survival curves (GraphPad Software, San Diego, CA, USA). *p* < 0.05 was considered to be statistically significant.

## Figures and Tables

**Table 1:** Primer sequences for semiquantitive Real-Time PCR.

Primer	Sequence
Actin	for: AGCCATGTACGTAGCCATCC, rev: CTCTCAGCTGTGGTGGTAA.
Havcr1	for: AAA CCA GAG ATT CCC ACA CG rev: GTCGTG GGT CTT CCT GTA GC
Acta2	for: GTG CTA TGT CGC TCT GGA CTT TGA rev: ATG AAA GAT GGC TGG AAG AGG GTC
Col1a1	for: GTCCCAACCCCCAAAGAC rev: CCCTCGACTCCTACATCTTCTGA
AZGP1	for: ATG GTG CCT GTC CTG CTG TC rev: TCG CAA CCA AAC ATT CCC TG
Ppargc1a	for: TAT GGA GTG ACA TAG AGT GTG CT rev: CCA CTT CAA TCC ACC CAG AAA G
Cpt1	for: CTC CGC CTG AGC CAT GAA G rev: CAC CAG TGA TGA TGC CAT TCT
Ppara	for: TGC AAA CTT GGA CTT GAA CG rev: GAT CAG CAT CCC GTC TTT GT
